# Influence of Human Telomerase Reverse Transcriptase Mutation on the Aggressiveness and Recurrence in Meningiomas

**DOI:** 10.7759/cureus.15342

**Published:** 2021-05-31

**Authors:** Balkan Sahin, Salim Katar, Saime A Şahin, Serdar Çevik, Sevket Evran, Oguz Baran, Canan Tanık, Hüseyin U Adılay, Adem Yılmaz

**Affiliations:** 1 Department of Neurosurgery, Sisli Hamidiye Etfal Research and Training Hospital, Istanbul, TUR; 2 Department of Neurosurgery, Medical Faculty of Balikesir University, Balıkesir, TUR; 3 Department of Neurosurgery, Memorial Sisli Hospital, Istanbul, TUR; 4 Department of Neurosurgery, Haseki Research and Training Hospital, Istanbul, TUR; 5 Department of Neurosurgery, Koç University, Istanbul, TUR; 6 Department of Pathology, Sisli Hamidiye Etfal Research and Training Hospital, Istanbul, TUR

**Keywords:** meningioma, telomerase reverse transcriptase, malignant progression, recurrent meningioma, tert promoter

## Abstract

Background: Over 200 human telomerase reverse transcriptase (hTERT) polymorphism combinations have been implicated in the development of cancer. This study aimed to evaluate hTERT mutations in meningioma tissue and its association with meningioma.

Material and Methods: A total of 90 patients who underwent surgery between 2006 and 2015 and were histopathologically diagnosed with meningioma (WHO 2016) were included.

Results: Among the 90 participants included herein, 50 (55.5%) and 40 (44.5%) were female and male, respectively, with an average age of 56.2 ± 14 years. Mean Ki-67 values were 10.56% (SD 12.41, range 0-60), while the mean follow-up duration was 39.1 months (SD 26.3). Low- and high-grade patients had a mean Ki-67 score of 4.31% (SD 3.58, range 0-16) and 19.92% (SD 14.91, range 2-60) (p = 0.0001). Our results showed a moderate positive correlation between Ki-67 score and the presence of hTERT mutation (Pearson correlation test, r = 0.5161; p = 0.0001). Patients with an hTERT mutation > 30% had significantly higher risk for reoperation than those with lower levels of mutation (p = 0.016, chi square test). None of the patients requiring reoperation had an hTERT mutation < 10%. Moreover, high-grade patients had a 7.2 times higher risk of reoperation than those with an hTERT mutation > 30%.

Conclusion: The presence of hTERT mutation, in addition to high Ki-67, indicated a more aggressive meningioma disease course and potentially increased risk of recurrence.

## Introduction

Meningiomas, one of the common central nervous system (CNS) tumors that account for 30% of all intracranial tumors, originate from arachnoidal cap cells along the dura mater, generally progress slowly, and are mostly benign [1]. These tumors have been classified into three groups by the World Health Organization (WHO). Among the meningiomas with a documented WHO grade, 81.1% are grade I (typical), 16.9% are grade II (atypical), and 1.7% are grade III (anaplastic) [2]. Some of the meningiomas have higher relapse rates and a more aggressive clinical course. According to histological grade, WHO grade I, II, and III tumors have a recurrence risk of 7%-25%, 29%-59%, and 60%-94%, respectively [3]. Therefore, both histopathological grading and cytological evaluations are important. Surgery is usually the first treatment option, but radiosurgery may become the first option in meningiomas that are deeply located and invade the vascular and neuronal structures [4].

Meningiomas can be caused by both internal and external factors. External factors include radiation, head injuries, and viruses, whereas internal factors comprise hormones and genetic predisposition. Evidence has shown that dysfunctions in specific genes may play a role in the formation of meningiomas. Tumor formation can be caused by the inactivation of tumor suppressor genes and overexpression of oncogenes. Apart from chromosome 22 deletions, which are the most common abnormality detected in meningiomas, losses in chromosomes 1p, 3p, 6q, 10q, and 14q have been found especially in atypical and malignant meningiomas [5,6]. Moreover, recent studies have shown that miRNA21-107 is closely related to grade changes, while MIR17HG is closely related to recurrence [7,8]. These genetic parameters have been proven useful as markers for tumor grade and recurrence. The primary aim of the aforementioned studies, especially those on atypical, malignant, and recurrent meningiomas, is to obtain information regarding appropriate treatment protocols and the course of the disease.

Telomerase reverse transcriptase (TERT; hTERT in humans) is an important catalytic subunit of the telomerase enzyme complex, together with the telomerase RNA component [9]. The hTERT gene consists of 16 exons and 15 introns on chromosome 5. The hTERT promoter is rich in GC and lacks the TATA and CAAT boxes but contains positions for many transcription factors, indicating high levels of regulation by multiple factors across many cellular components [10].

Studies on the hTERT gene have focused on its mutation and relationship with the risk of cancer. Over 200 hTERT polymorphism combinations have been implicated in the development of cancer [11], while hTERT regulation has been investigated to determine the potential mechanisms of telomerase activation in cancer cells. Moreover, glycogen synthase kinase 3 (GSK3) seems to be overexpressed in most of the cancer cells [12]. The current study aimed to evaluate hTERT mutation in meningioma tissue and determine its association with meningioma.

## Materials and methods

Between 2006 and 2015, 90 patients who underwent surgery at the neurosurgery clinic of the University of Health Sciences, Sisli Hamidiye Training and Research Hospital and were histopathologically diagnosed with meningioma were included herein. Sociodemographic data and clinical characteristics of the patients were recorded. Pathologic classification was made according to the WHO 2016 system. This study was approved by the ethics committee of Şişli Hamidiye Training and Research Hospital. All human data used herein were collected according to the principles of the aforementioned ethics committee. Informed consent was obtained from each patient before study participation.

A total of 110 tumor tissues from 90 patients were used to create paraffin blocks. Thereafter, paraffin blocks were sectioned at a thickness of 4 µm on the slide and incubated at 60 °C for 45-60 min until the paraffin on the slide melted. The sections were then placed into the LEICA Bond Max device and stained.

 Patients included herein were divided into two groups according to WHO 2016 criteria: low-grade (LG) patients (those with grade I disease) and high-grade (HG) patients (those with grade II and III disease).

 The technical characteristics of the method used for the primary antibody were as follows: manufacturer, EMD Millipore; product code, ABE2075; antibody name, TERT (Polyclonal); operating dilution, 1:100; antigen retrieval method, LEICA ER1 (Citrate) 30 min; antibody incubation time, 1 h; used device, LEICA Bond Max. The technical characteristics of the method used for the secondary kit were as follows: manufacturer, LEICA; product code, DS9800; kit name, Bond Polymer Refine Detection DAB.

**Figure 1:**
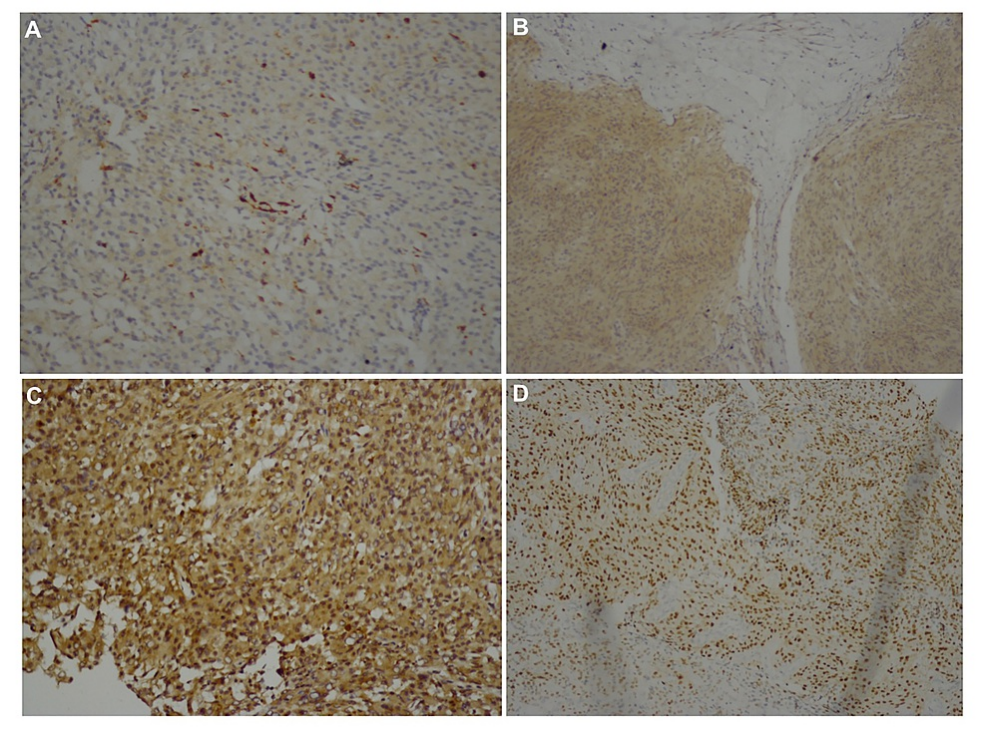
The presence of anti-hTERT antigen in immunohistochemical staining (A) TERT ×100, one-positive. (B) TERT ×100, two-positive. (C) TERT ×200, three-positive. (D) TERT ×100, four-positive.

 The hTERT index was assessed using an image analyzer (Lucia 32G/Mutech; Nikon, Japan) in 10 fields (×20 lens) and semi-quantitatively graded for anti-TERT antigen through immunohistochemical staining expressed as the percentage of positive cells. All patients were classified into five different groups according to the presence and percentage of hTERT-labeled cells. A zero positive value was given when no stained cell was present. Moreover, a staining rate of 1%-10% was evaluated as one-positive; 11%-30% as two-positive, 31%-60% as three-positive, and 61% and more as four-positive [13]. This study examined the presence of anti-hTERT antigen in immunohistochemical staining (Figures 1A-1D).

All statistical analyses were performed using SPSS version 11 software (SPSS Inc., Chicago, IL, USA). The normality of the variable distribution was examined using visual and analytical methods (Kolmogorov-Smirnov/Shapiro-Wilk tests). Normally distributed descriptive variables were presented as means and standard deviations. Parameters in 2 × 2 cross-tabulations were compared using the Pearson, chi-square, and Fisher exact tests. Bonferroni correction and post-hoc analyses were performed in more than 2 × 2 tabulations. A 5% overall probability for a type I error was used to determine statistical significance.

## Results

A total of 90 patients were included herein. All meningiomas were Simpson Grade I to III resected. Reoperation was performed in 16 (17.8%) patients, among whom 12 and four underwent two and three surgeries, respectively. Among the 90 patients, 54 (60%), 28 (31.1%), and eight (8.9%) had grades I, II, and III diseases, respectively (Table 1).

**Table 1:** Characteristic features of the 90 patients who underwent surgical decompression for meningioma treatment

Sex	n (%)
Female	50 (55.5%)
Male	40 (44.5%)
WHO grade	n (%)
Grade I	54 (60%)
Grade II	28 (31.1%)
Grade III	8 (8.9%)
Tumor localization	n (%)
Anterior fossa	7 (7.7%)
Middle fossa	6 (6.6%)
Posterior fossa	11 (12.2%)
* *Convexity	
Frontal	17 (18.8%)
Frontoparietal	5 (5.5%)
Parietal	15 (16.6%)
Temporal	14 (15.5%)
Temporoparietal	4 (4.4%)
Occipital	2 (2.2%)
Parieto-occipital	5 (5.5%)
Parasagittal	4 (4.4%)
Histological subtype	n (%)
Angiomatous	4 (4.4%)
Fibrous	4 (4.4%)
Transitional	6 (6.6%)
Psammomatous	3 (3.3%)
Meningothelial	35 (38.8%)
Lymphoplasmacyte-rich	1 (1.1%)
Secretory	1 (1.1%)
Atypical	24 (26.6%)
Chordoid	2 (2.2%)
Anaplastic	9 (9.9%)
Papillary	1 (1.1%)
Tumor size	n (%)
<3 cm	9 (10%)
3–5 cm	40 (44.4%)
>5 cm	41 (45.6%)

Among the 16 patients who underwent reoperation, two had grade I disease (tumor locations; frontal and temporal), 10 had grade II disease (tumor locations; one frontoparietal, four parietal, three anterior fossa, and two parieto-occipital), and four had grade III disease (tumor locations; one frontal, one temporal, and two parietal). Grade change was observed in five patients (31.25%) who underwent reoperation (resulting from a relapse), among whom three exhibited an increase from grade II to grade III, whereas two exhibited a decrease from grade I to grade II. Locations, sizes, and histopathological subtypes are summarized in Table 1. Among the included patients, 50 (55.5%) and 40 (44.5%) were female and male, respectively, with a mean age of 56.2 (SD 14). The mean Ki-67 value was 10.56% (SD 12.41, range 0-60), while the mean follow-up duration was 39.1 months (SD 26.3). The LG group had an average Ki-67 score of 4.31% (SD 3.58, range 0-16, whereas the HG group had an average Ki-67 score of 19.92% (SD 14.91, range 2-60) (p = 0.0001). No significant differences in gender and age were observed between the groups. Moreover, no significant differences in tumor location, tumor size, Ki-67 scores, and the presence of hTERT mutation were noted between both groups (p = 0.262, 0.395, 0.694, and 0.933, respectively) (Kruskal-Wallis test). Patients with an hTERT mutation < 10% had an average Ki-67 score of 4.13% (SD 3.42, range 0-12), whereas those with an hTERT mutation > 10% had an average score of 17.27% (SD 14.71, range 2-60) (p = 0.0001, t-test). Among the LG group, patients with an hTERT mutation < 10% had a mean Ki-67 score of 3.54% (SD 2.99, range 0-12), whereas those with an hTERT mutation > 10% had an average score of 6.77% (SD 4.28, range 2-16) (p = 0.008, Mann-Whitney U test). Among the HG group, those with an hTERT mutation < 10% had an average Ki-67 score of 9% (SD 2.92, range 5-12), whereas those with an hTERT mutation > 10% had an average score of 21.68% (SD 15.33, range 5-60) (p = 0.031, Mann-Whitney U test) (Table 2).

**Table 2:** Comparison of Ki-67 scores between patient groups. hTERT, human telomerase reverse transcriptase HG, High-grade

	Ki-67			
All patients (n = 90)	Mean	SD	Range	P-value
hTERT ≤ 10%	4.13%	3,42	0-12	0.0001
hTERT > 10%	17.27%	14.71	2-60	
Low-grade (n = 54)				
hTERT ≤ 10%	3,54%	2,99	0-12	0.008
hTERT > 10%	6,77%	4,28	2-16	
High-grade (n = 36)				
hTERT ≤ 10%	9%	2,92	5-12	0.031
hTERT > 10%	21.68%	15.33	5-60	
Reoperation (+) HG (n = 14)	22.14%	13.74	5-50	0.401
Reoperation (-) HG (n = 12)	−	15.61	2-60	

A moderate positive correlation between Ki-67 score and the presence of the hTERT mutation was observed (Pearson correlation test, r = 0.5161; p = 0.0001). HG patients requiring reoperation had an average Ki-67 score of 22.14% (SD 13.74, range 5-50), where HG patients no requiring reoperation had an average score of 19.73% (SD 15.61, range 2-60) (p = 0.401) (Table 2). Patients with an hTERT mutation > 30% had a significantly greater risk of reoperation than those with lower levels of mutation (p = 0.016, chi-square test). None of the patients requiring reoperation had an hTERT mutation < 10%. Moreover, among the HG group, patients with an hTERT mutation > 30% had a 7.2 times higher risk of needing reoperation.

## Discussion

Telomerases are a different subgroup of RNA-dependent polymerases. The enzyme, which uses the RNA it carries as a template and ensures the preservation of telomere ends shortened by every division, adds telomeric DNA sequences to the ends of linear chromosomes. Given that DNA polymerases cannot initiate new DNA synthesis at the 3′ prime in the main chain, the length of a chromosome is shortened after each DNA doubling. Telomerases address this prime replication problem by inserting telomeric repeat sequences at the 3′ prime of the chromosome, consequently hindering the shortening of the chromosome [14]. Given the small number of telomerases in somatic cells, a typical cell shortens its telomere length by about 100 base pairs at each division. This telomere shortening gradually leads to the cessation of the cellular division [9]. As such, the majority of the molecular genetic studies being performed today aim at addressing “telomere-induced cell aging.” TERT is an important catalytic subunit of the telomerase enzyme complex, together with the telomerase RNA component [9]. In particular, TERT functions to catalyze the incorporation of TTAGGG nucleotides into telomeres on chromosomal ends [14]. As reported in cell culture studies, hTERT renders normal cells immortal by offering the self-renewal property of stem cells to other cells [15]. Moreover, studies haves shown that hTERT overexpression is often associated with cancers and tumor formation [16]. Research regarding the hTERT gene has focused on its mutation and relationship with the risk of cancer. Over 200 polymorphism combinations of hTERT have been found to be involved in cancer development [11]. Moreover, studies have investigated hTERT regulation to determine possible mechanisms of telomerase activation in cancer cells. Evidence has shown that GSK3 appears to be overexpressed in most cancer cells and that leptin increases the mRNA expression of hTERT through signal transducer and activators of transcription 3, suggesting a mechanism for an increased incidence of cancer in obese individuals [12]. Together with CTNNB1 mutations, hTERT mutations have been shown to be associated with hepatocellular carcinomas and adenomas [17]. Studies investigating the potential involvement of hTERT mutations in malignant transformation have suggested an association between hTERT mutations and meningioma. Similarly, TERT promoter mutations have been detected in low-grade tumors, suggesting that TERT promoter mutations may occur early during tumor development, may play a role in the formation of meningiomas predisposes to malignant progression, and may promote the survival of cells with chromosomal imbalances [18]. Nevertheless, insufficient data have been available on the effects of hTERT mutations on meningioma prognosis, except for those reported in high-grade and recurrent meningiomas. The current study found a moderate positive correlation between the Ki-67 score and the presence of hTERT mutation, which may explain the transformation of low-grade meningiomas.

**Figure 2:**
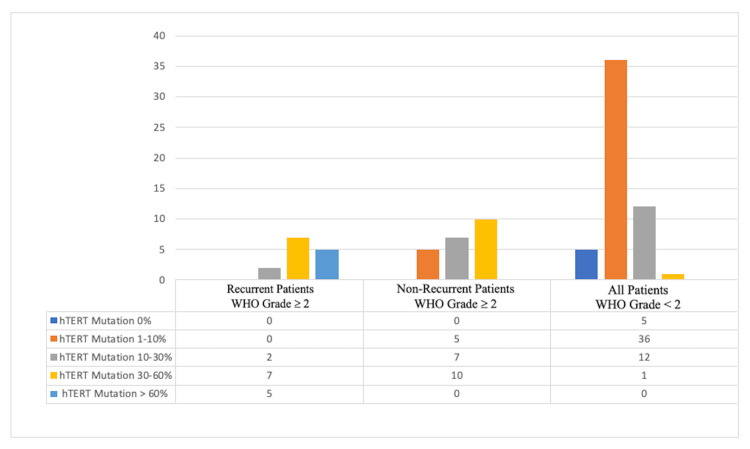
hTERT staining percentages in recurrent patients with WHO grade ≥ II, non-recurrent patients with WHO grade ≥ II, and all patients with WHO grade < II disease. hTERT, human telomerase reverse transcriptase

The study by Koelsche et al. examined TERT promoter mutations in 91 subjects with WHO grade I, 49 subjects with grade II, and 37 subjects with grade III meningiomas. Although the investigators detected no mutation in grade I meningiomas, 4% and 16% of subjects with grade II and III meningiomas had TERT promotor mutations, respectively [19]. Goutagny et al. found that grade II and III meningiomas had higher hTERT expression than grade I meningiomas after determining the association between hTERT mutations and malignant transformation through DNA and RNA analysis of 85 frozen tumor tissues from 73 patients [20]. After performing a multi-center study on 252 meningioma cases (119, 88, and 45 WHO grade I, II, and III cases, respectively), Sahm et al. concluded that TERT promoter mutations could be helpful in meningioma grading and causes increased meningioma grades and early recurrences [21]. The current study found that patients with an hTERT mutation > 30% had a significantly higher risk of reoperation than those with lower mutation levels. Moreover, none of the patients requiring reoperation had an hTERT mutation < 10%. Among the HG group, those with hTERT mutation > 30% had a 7.2 times higher risk of reoperation (Figure 2).

A study published in 2015 by Abedalthagafi et al. suggested that hTERT mutations and its subtypes could provide new insights into intra-tumoral heterogeneity in the pathways of malignant progression and into the evolution of malignant phenotypes in anaplastic meningiomas [22]. The findings obtained in the current study are consistent with those presented in the literature and support available data.

Our study has several limitations word noting. First, hTERT mutation was evaluated through immunohistochemistry. Moreover, our data could be regarded as less reliable than DNA or RNA analysis given that hTERT antigen staining is a semiquantitative assessment that is expressed in percentages. Considering that our study did not feature a healthy control group, hTERT mutation was compared using several independent variables, including histopathologic grade in particular.

## Conclusions

The current study showed that the presence of hTERT mutations in addition to high Ki-67 suggests a more aggressive meningioma course and potentially increased risk of recurrence. As such, more aggressive surgical strategies and early adjuvant radiotherapy may be required. We believe that hTERT mutations will become an important biomarker that could significantly impact our clinical approach to the planning and management of meningiomas, which needs to be confirmed by further studies.

## References

[1] Marosi C, Hassler M, Roessler K, Reni M, Sant M, Mazza E, Vecht C (2008). Meningioma. Crit Rev Oncol Hematol.

[2] Ostrom QT, Gittleman H, Xu J, Kromer C, Wolinsky Y, Kruchko C, Barnholtz-Sloan JS (2016). CBTRUS Statistical Report: primary brain and other central nervous system tumors diagnosed in the United States in 2009-2013. Neuro Oncol.

[3] Marciscano AE, Stemmer-Rachamimov AO, Niemierko A (2016). Benign meningiomas (WHO Grade I) with atypical histological features: correlation of histopathological features with clinical outcomes. J Neurosurg.

[4] Prabhu VC, Melian E, Germanwala AV, Solanki AA, Borys E, Barton K, Anderson DE (2018). Cranial base meningiomas. World Neurosurg.

[5] Mawrin C, Perry A (2010). Pathological classification and molecular genetics of meningiomas. J Neurooncol.

[6] Zang KD (2001). Meningioma: a cytogenetic model of a complex benign human tumor, including data on 394 karyotyped cases. Cytogenet Cell Genet.

[7] Evran S, Baran O, Kayhan A (2020). The expression of MIR17HG protein as a potential therapeutic target in meningioma. World Neurosurg.

[8] Katar S, Baran O, Evran S (2017). Expression of miRNA-21, miRNA-107, miRNA-137 and miRNA-29b in meningioma. Clin Neurol Neurosurg.

[9] Weinrich SL, Pruzan R, Ma L (1997). Reconstitution of human telomerase with the template RNA component hTR and the catalytic protein subunit hTRT. Nat Genet.

[10] Cong YS, Wen J, Bacchetti S (1999). The human telomerase catalytic subunit hTERT: organization of the gene and characterization of the promoter. Hum Mol Genet.

[11] Mocellin S, Verdi D, Pooley KA (2012). Telomerase reverse transcriptase locus polymorphisms and cancer risk: a field synopsis and meta-analysis. J Natl Cancer Inst.

[12] Sundin T, Hentosh P (2012). InTERTesting association between telomerase, mTOR and phytochemicals. Expert Rev Mol Med.

[13] Mandrioli L, Panarese S, Cesari A, Mandara MT, Marcato PS, Bettini G (2007). Immunohistochemical expression of h-telomerase reverse transcriptase in canine and feline meningiomas. J Vet Sci.

[14] Shampay J, Blackburn EH (1988). Generation of telomere-length heterogeneity in Saccharomyces cerevisiae. Proc Natl Acad Sci USA.

[15] Cukusić A, Skrobot Vidacek N, Sopta M, Rubelj I (2008). Telomerase regulation at the crossroads of cell fate. Cytogenet Genome Res.

[16] Flores I, Benetti R, Blasco MA (2006). Telomerase regulation and stem cell behaviour. Curr Opin Cell Biol.

[17] Nault JC, Mallet M, Pilati C (2013). High frequency of telomerase reverse-transcriptase promoter somatic mutations in hepatocellular carcinoma and preneoplastic lesions. Nat Commun.

[18] Goutagny S, Yang HW, Zucman-Rossi J (2010). Genomic profiling reveals alternative genetic pathways of meningioma malignant progression dependent on the underlying NF2 status. Clin Cancer Res.

[19] Koelsche C, Sahm F, Capper D (2013). Distribution of TERT promoter mutations in pediatric and adult tumors of the nervous system. Acta Neuropathol.

[20] Goutagny S, Nault JC, Mallet M, Henin D, Rossi JZ, Kalamarides M (2014). High incidence of activating TERT promoter mutations in meningiomas undergoing malignant progression. Brain Pathol.

[21] Sahm F, Schrimpf D, Olar A (2016). TERT promoter mutations and risk of rcurrence in meningioma. J Natl Cancer Inst.

[22] Abedalthagafi MS, Bi WL, Merrill PH (2015). ARID1A and TERT promoter mutations in dedifferentiated meningioma. Cancer Genet.

